# High prevalence of strabismic visual field expansion in pediatric homonymous hemianopia

**DOI:** 10.1371/journal.pone.0209213

**Published:** 2018-12-19

**Authors:** P. Matthew Bronstad, Eli Peli, Rui Liu, Amy Doherty, Anne B. Fulton

**Affiliations:** 1 Schepens Eye Research Institute, Massachusetts Eye and Ear, Boston, Massachusetts, United States of America; 2 Department of Ophthalmology, Harvard Medical School, Boston, Massachusetts, United States of America; 3 Boston Children’s Hospital, Boston, Massachusetts, United States of America; Faculty of Medicine, Cairo University, EGYPT

## Abstract

If homonymous hemianopia develops in childhood it is frequently accompanied by strabismus. In some of these cases the strabismus increases the size of the binocular visual field. We determined how prevalent visual-field-expanding strabismus is in children who have homonymous hemianopia. Medical records were examined from 103 hemianopic patients with exotropia (XT) or esotropia (ET). For each participant, we determined whether their strabismus was in a direction that resulted in visual field expansion (i.e. left exotropia with left homonymous hemianopia). Ages at which hemianopia and strabismus were first noted were compared to determine which developed first. The prevalence of XT (24%) and ET (9%) with homonymous hemianopia were both much higher than in the general population (1.5% and 5%, respectively). More strabismic eyes pointed to the blind than seeing side (62 vs 41, 60% vs. 40%, *p* = 0.02). Exotropic eyes were five times more likely to point to the blind side than esotropic eyes (85% vs 15%). Strabismus, especially exotropia, is much more common in pediatric homonymous hemianopia than in the general population. The strabismus is significantly more often in a visual field-expanding direction. These results support an adaptive role for the strabismus. Patients with HH and exotropia or esotropia should be aware that their visual field could be reduced by strabismus surgery.

## Introduction

**Fig 1:**
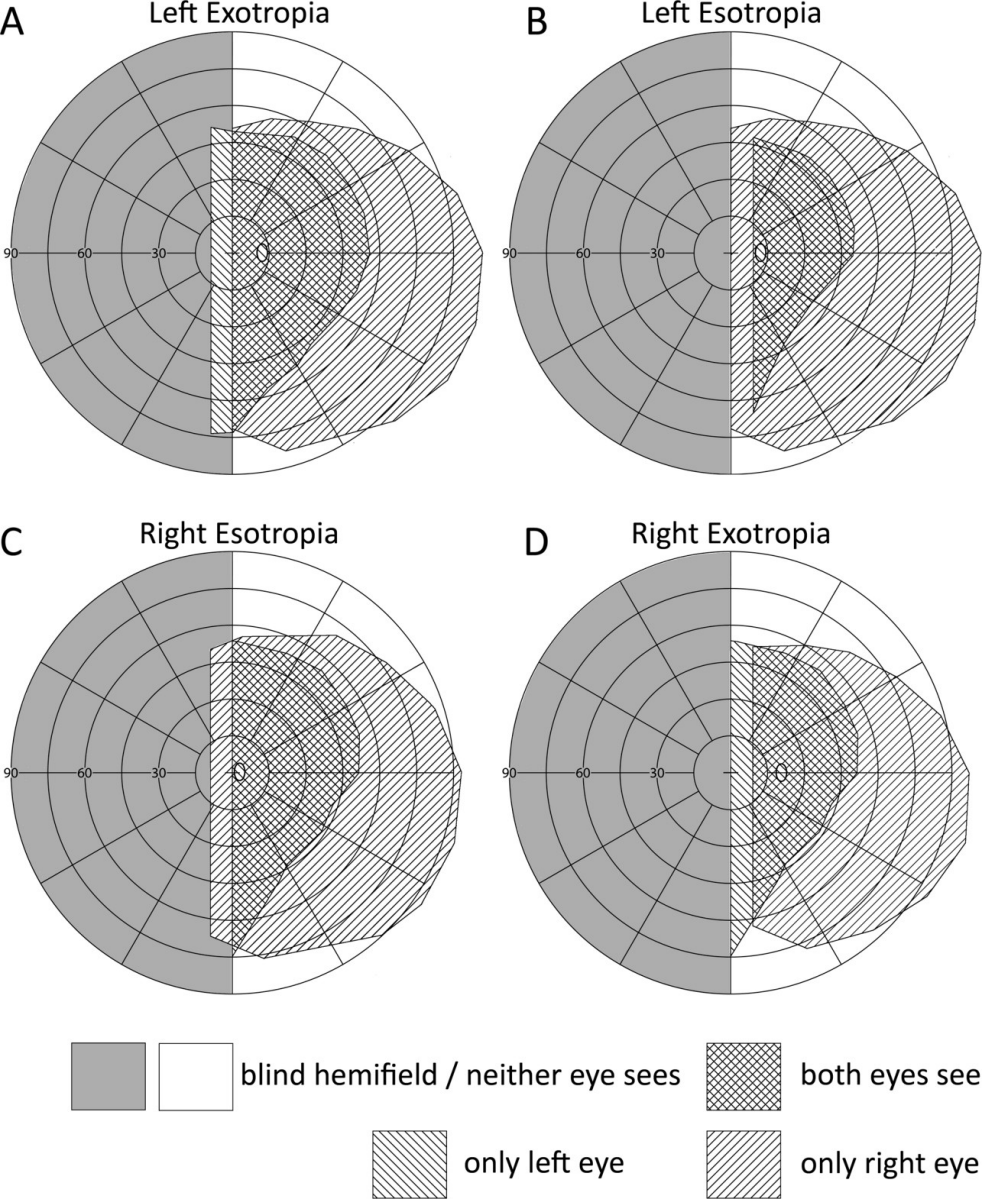
Visual fields of the four possible combinations of left HH and strabismus, constructed from interpolated plots of monocular observers. [28] (Top row) The top plots depict visual fields when the right eye fixating and in primary position of gaze, while the left eye is fixating in the two bottom plots. (A) Left XT of 12° (20 prism diopters) expands the central field of view left of the vertical meridian. (C) Right ET of 12° expands the central field of view to the left similarly but reduces the field of view at the far right periphery. This may be beneficial despite no net gain in horizontal field span. (B&D) In left ET and right XT there is no visual field expansion. The small reduction on the right temporal field is due to obstruction by the orbit. In all cases large regions of the visual field, including the central view may be diplopic and/or affected by binocular visual confusion. Esotropia causes contralateral monocular peripheral visual field reduction (B, left eye) due to the center of eye rotation behind the pupil. This causes the nose to block more of peripheral nasal field as the eye adducts. Conversely, exotropia (A, left eye) causes monocular peripheral expansion nasally.

Homonymous hemianopia (HH), the loss of one half of the visual field on the same side in both eyes, results from stroke, tumor, trauma, or surgery.[1] It has been suggested that strabismus may compensate for hemianopia.[2–8] In HH, exotropia of the eye on the side of the field loss provides binocular visual field expansion equal to the angle of strabismic deviation (Fig 1A). With esotropia of the eye contralateral to the field loss, binocular field expansion is also possible[9] but at the expense of reduction of far peripheral field on the seeing side (Fig 1C). While this field substitution does not increase the total horizontal extent of the field of view,[10] the benefits of an expanded central visual field outweigh the associated loss in far peripheral field. Suppression and anomalous retinal correspondence, both of which develop more readily in children, may circumvent the central double vision inherent in misaligned eyes.[11–13] The same strabismus in adults with HH is expected to cause intractable double vision which may counter the benefit of visual field expansion.

**Fig 2:**
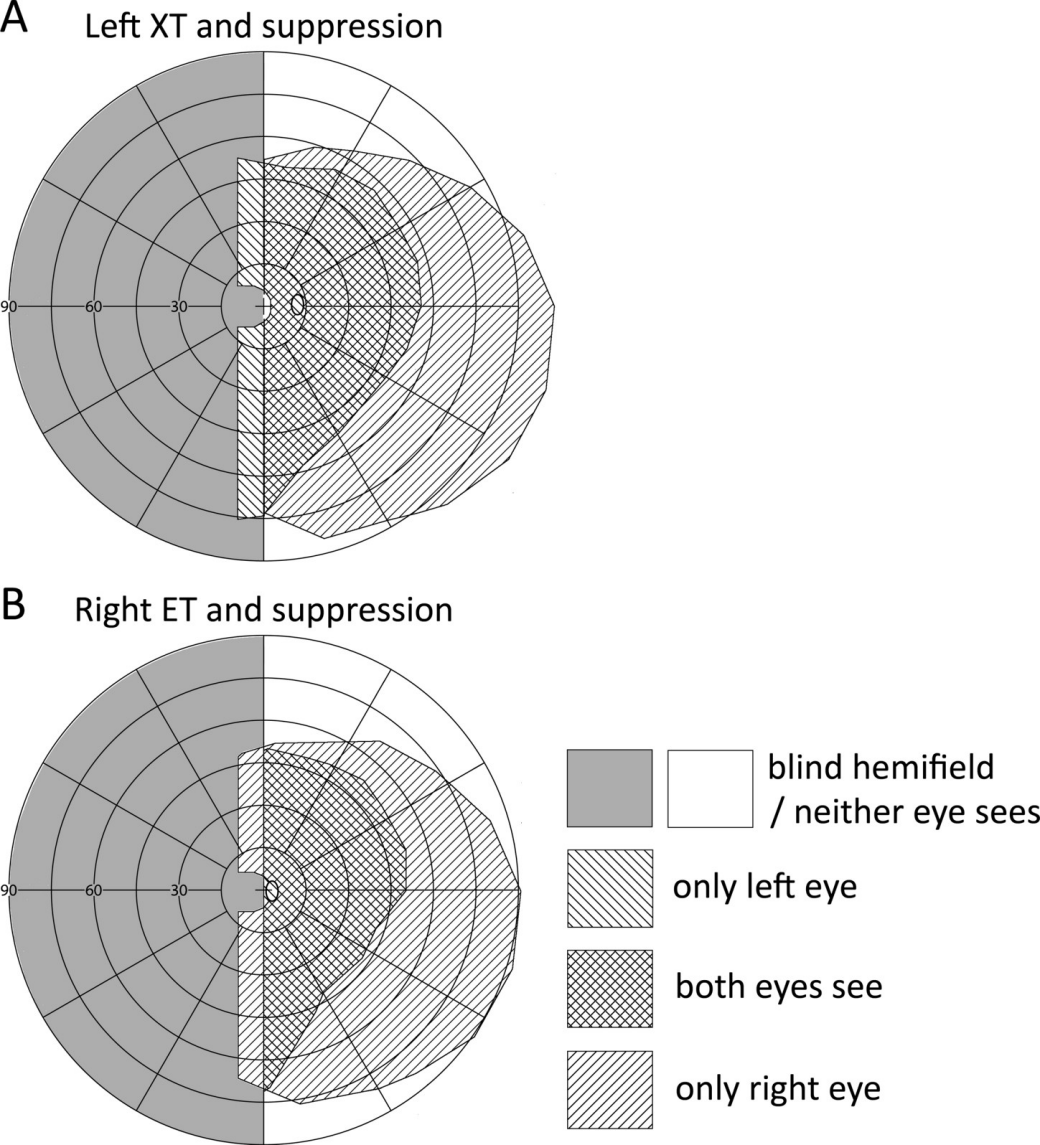
Two cases in which central suppression[29] makes strabismic field expansion more tolerable by eliminating central diplopia. (B) Proximity of the deviating eye’s physiological blind spot to the fixing eye’s fovea further expands the central area that is free of double vision.

There are large regions of potential double vision in all plots in Fig 1. Visual field expansion is possible in the cases depicted in Fig 1A and 1C) even in the presence of central suppression (Fig 2). Suppression reduces the impact of visual confusion and diplopia centrally while peripheral visual field expansion above and below the horizontal midline is still available. Diplopia and confusion are more readily tolerated in the peripheral visual field.[14]

Two recent studies showed that HH following hemispherectomies often results in field expanding exotropias.[8, 15] Exotropia also occurs after stroke and other brain injuries[16] that cause hemianopia,[17, 18] but the prevalence of field-expanding strabismus with HH, due to causes other than hemispherectomy, is unknown; only a handful of cases, mostly young adults with presumed long standing HH, were reported in the literature.[2–8] If horizontal strabismus is an adaptation that compensates for field loss, then more patients with HH, due to any cause, should have the strabismic eye pointing to the blind rather than the seeing side, as long as the strabismus did not precede the HH. The prevalence of field-expanding esotropia (Fig 1C) is also unknown and the effect has been rarely if ever reported.

We reviewed medical records at Boston Children’s Hospital to estimate the prevalence of strabismus in a population of patients with early onset of HH, and compare the prevalence of para-central field expansion afforded by strabismus.

## Methods

### Ethics statement

The research adhered to the tenets of the Declaration of Helsinki. The Boston Children’s Hospital (BCH) IRB approved the study, waiving informed consent for review of records, and reporting of anonymized data.

### Medical record review

We searched for patients with hemianopia and esotropia and those with hemianopia and exotropia. The records were reviewed to determine the side of the visual field defect, which eye deviated, the direction of the horizontal deviation—esotropic or exotropic—to determine whether the strabismus offered visual field expansion. For example, in left HH, left XT is field expanding because the strabismic eye points into the blind side. The ages at which HH and strabismus were first documented, visual acuities, and refractive error were also noted. Cranial nerve palsy involvement was documented if present.

At early ages, the HH was documented by confrontation testing performed by experienced pediatric neurologists or ophthalmologists. Although the majority of patients were too young for perimetry at the time of diagnosis of HH, the hemifield deficits were eventually characterized by perimetry n = 91 (e.g., Goldmann, Humphrey Field Analyzer); only results of confrontation testing were available in 12. Visual acuity was measured using symbols (Snellen, LEA symbols, Sheridan Gardiner letters) n = 75, or gratings by preferential looking using Teller acuity cards,[19] n = 24. Acuity was not recorded in 4 cases. Retinal correspondence had been evaluated in only 24 cases. Clinical evaluation of suppression was measured in 24 of the 103 using the Worth 4-dot test, it was attempted in an additional 9 but could not be measured due to limited ability to participate in sensory testing, and was not mentioned in 70.

Nonparametric tests were used as appropriate for inferential statistical comparisons between groups.

## Results

Basic demographics are provided in Table 1.

**Table 1:** Basic demographics.

Sex	Female = 47, Male = 56
Hemianopia	Right = 51, Left = 52
Strabismus	XT = 75, ET 28
Age at first exam	3.1 years (0.2–23.9)
Acuity, better/worse eye	0.42/0.66 LogMAR (average)

### 1. Prevalence of strabismus in children with HH

The database search identified 396 patients with hemianopia; 142 were coded as being also exotropic or esotropic. We excluded those who had no light perception in the strabismic eye or strabismus was not well documented n = 11 (8%). In 28 of the 142 (19.7%), documentation of HH was not found or hemianopia was altitudinal or bitemporal. The remaining 103, who were confirmed to have homonymous hemianopia and strabismus, were retained for record review and further analyses.

### 2. Direction of strabismus. Do strabismic eyes point to the blind side?

**Fig 3:**
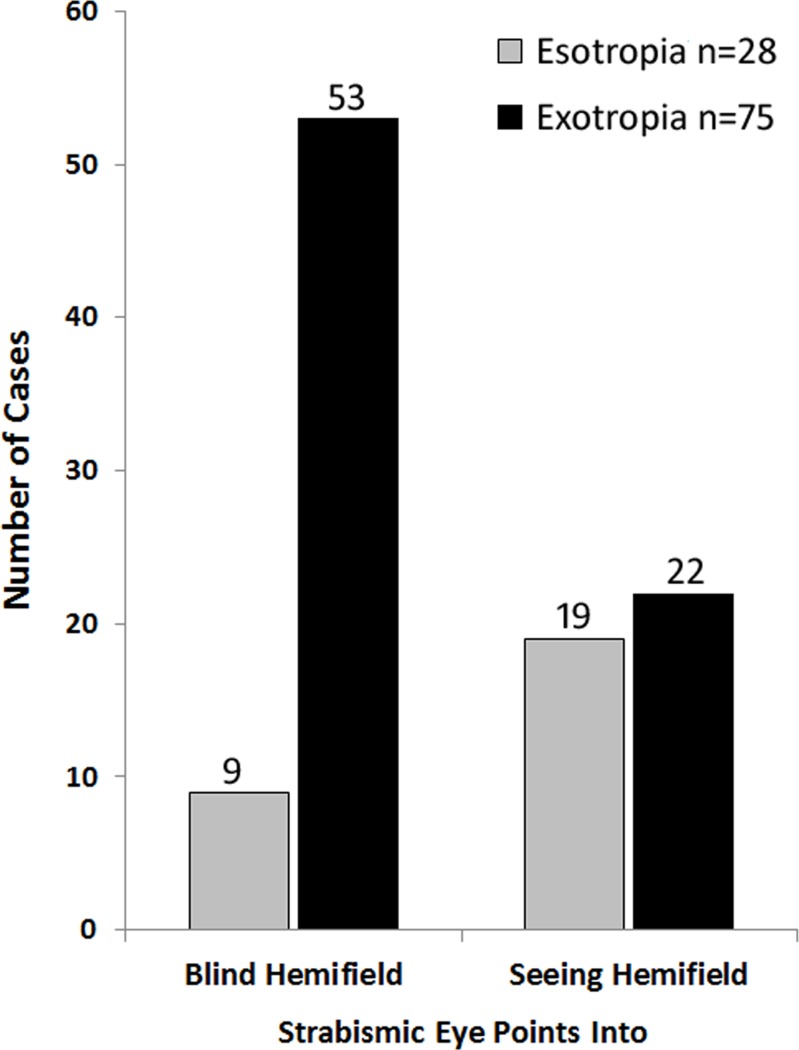
The number of cases in which esotropic or exotropic eyes pointed to the blind or seeing sides (out of a total of 103).

There were substantially more XT (n = 75) than ET eyes (n = 28). Fig 3 shows the number of exotropic (XT) and esotropic (ET) eyes pointing to the blind and seeing sides. Sixty-two strabismic eyes pointed to the blind side, which is statistically significant (binomial *p* = 0.02). Strabismic deviations into the blind side (field of view expanding) were much more commonly XT (n = 53; 71%) than ET (n = 9; 32%), whereas deviations to the seeing side were much more commonly ET (n = 19; 68%) than XT (n = 22; 29%). These differences were significant, *χ*^2^ = 17.97, *p* = 0.00002.

The proportion of strabismic eyes pointing to the blind side varied little by cause of field loss: periventricular leukomalacia and hypoxic ischemic encephalopathy, (11/16 = 69%); stroke, 31/49 = 63%; hemispherectomy for seizures, 10/17 = 59%; tumor resection, 8/15 = 53%; trauma, 2/6 = 33%.

### 3. Prevalence of strabismus in children with HH

The estimate of the prevalence of strabismus in children with HH is based on the assumption that the frequency of unverified HH in the larger population of 396 was the same as in the subgroup of 142 who had strabismus. Specifically, 318 = 396*(100–19.7%, *i*.*e*., removing the same percentage of unverified HH) was taken as the denominator. Compared to the percentage of XT and ET in the general population (1.5% and 5%,^18,19^ respectivey), XT and ET occur significantly more often in this population with HH (XT: 75/318 or 23.6% vs. 1.5%, binomial *p*<0.0001; ET: 28/318 or 8.8% vs 5%, binomial *p* = 0.003), respectively.

### 4. Sequence of strabismus and hemianopia diagnoses

For the 103 participants, the median age at first eye examination was 3.1 years (range 0.2 to 23.9 y). Only two cases of strabismus, one XT, one ET, were reported to precede the visual field defect or the defect inducing disorder. The patient with XT was also notable as having a congenital third nerve palsy (the only one noted in the population of 103) that preceded the HH.

For those in whom a perinatal event was the basis for the hemianopia (n = 46), the strabismus was reported only at an older age: ET median 1.4 years (IQR = 0.75 to 3 y, range 0–14 y); XT median 4 years, (IQR = 1 to 5 y, range 0.25–17 y). The age difference between XT and ET only approached significance by Mann-Whitney U = 169, *p* = 0.08.

For those whose in whom the HH inducing event (stroke, tumor, trauma or surgery) occurred after the perinatal period (>7 days), the median age of onset of the HH was 1 year (IQR = 0.25 to 3y; range 0.04 to 15 y). Their median age of first reported ET was 1.5 year (IQR = 1 to 2y range 0 to 10y) and, of XT, 7 years (IQR = 4 y to 10 y range 0 to 25y), which was significantly different, Mann-Whitney U = 51.5, *p* = 0.02.

There was little difference in age of onset between those with left and right hemianopia (medians for LHH vs RHH = 0.04 vs 0 y, means = 1.5 vs 1.4 y range 0–13 vs 0–15 y). The ages of diagnosis of strabismus (medians 4 vs 3 y, means 5.3 vs 5.1 y; ranges 0.33 to 25 vs 0 to 17) were also similar.

### 5. Right versus left hemispheric damage

The proportion of right hemispherectomies (LHH: 7/12 cases) with left XT and left hemispherectomies (RHH: 3/5 cases) with right XT, was similar, *χ*^2^ = 0.001, *p* = 0.97. Patients with HH associated with causes other than hemispherectomy were more likely to have left XT with LHH: 24/40 (60%) than right XT with RHH: 20/46 (43%), but this was not significantly different, *p* = 0.29.

### 6. Visual acuities of strabismic and fellow eyes

Patients with a strabismic eye pointing to the blind side had mean LogMAR acuities of 0.61 (20/80) and 0.38 (20/47) in the strabismic and fellow eyes, respectively. For those with strabismic eyes pointing to seeing side, the acuities in the strabismic and fellow eyes were 0.74 (20/110) and 0.46 (20/58), respectively. The average difference in acuity between strabismic and fellow eyes was 0.25 LogMAR units. The difference in acuities between those with potential field expansion and those without was not significant.

## Discussion

In patients with early-onset HH we found that the prevalence of XT was 24%, and ET was 9%. These percentages are much higher than the 1.5% and 5%, respectively, reported in the general population.[18, 20] Moreover, HH almost always preceded strabismus, and strabismic eyes pointed to the blind side more frequently than expected by chance.

These findings support the hypothesis that the strabismus may be a compensatory adaptation to visual field loss. It is also noteworthy that among those with HH and strabismus the frequency of deviation to the blind side was much higher in XT (71%) than the frequency of deviating to the blind side in ET (32%). This is further support to the compensatory hypothesis, as XT provides actual field of view expansion while ET expands the central field but at the expense of similar reduction in the far peripheral field (Fig 1C). The large difference in prevalence between XT and ET may be one reason that ET has been rarely reported as comorbid with HH.[8, 21] The fact that it is not intuitive that esotropia may expand the field of view may have contributed to lack of reporting of ET as compensatory.

Nearly all strabismic deviations were reported later than the reported onset of HH. This temporal priority is also consistent with the hypothesis that strabismus may be an adaptation to HH. One may postulate that perinatal field loss encourages and exaggerates the tendency of infants to remain exotropic; 33% of newborns are XT, compared to 3% ET.[22] This explanation, however, is not supported by our data: four years is the median age at which exotropia was first noted in those with perinatal HH. However, in this retrospective study, the timing data are imperfect, depending on the accuracy of the reports obtained during the course of clinical care.

Anomalous retinal correspondence (ARC) is assumed to provide an optimal solution for strabismic field expansion in HH by eliminating the associated double vision through harmonization of the perceived visual directions in the eyes.[2] It is thought that ARC develops before age 7 years.[2, 13, 23] It is, therefore, curious that the onset of exotropia in our sample appears to occur at such a late age. We were unable to test the hypothesis that ARC had developed later as there was insufficient information about ARC in these retrospective data. ARC may develop later than previously thought, or field expansion may occur independent of ARC. Double vision may also be avoided by central suppression that preserves the expanded peripheral field (Fig 2).[24] It was recently reported that a woman with HH from traumatic brain injury at age 39 subsequently acquired XT that resulted in 30° of field expansion. She stated that she rarely perceived double vision and it did not trouble her when it was noticeable.[25]

The high frequency of strabismus in adults after brain injury[17] including stroke[26] warrants mention. Compared to a prevalence of 32% in our pediatric participants with HH, Fowler[17] reported strabismus in 37% (n = 89) of 239 adults with a history of brain injury from diverse causes. Similar to our findings, XT was much more common than ET. Moreover, Rowe[26] found 16.5% of 512 adults developed strabismus after strokes which was more often exotropia (72%). Of those with strabismus[26], a third had visual field loss, which was HH in 67%.

In the Koenraads et al[8] study of children with a history of hemispherectomy, those with right hemispherectomy (64% of 22) were more likely than those with left hemispherectomy (17% of 23) to develop exotropia. We found that those who had right hemispherectomies were equally likely as those with left hemispherectomies to result in contralateral exotropia. We also did not find any side bias with respect to field expanding tropias in our sample.

Two prospective studies showed that field expanding strabismus often follows hemispherectomy.[8, 15] Taken together with our findings, that exotropia develops considerably more often in this pediatric population with HH, and when it does it is more often field expanding, the hypothesis that juvenile hemianopia leads to visual field expanding strabismus is strongly supported.

Exotropic expansion may permit some patients to meet the legal requirements for a driving license in some states. Assuming a strabismic deviation of 25Δ the total horizontal extent of visual field would be 104 degrees. Nine US states and the District of Columbia have visual field requirements from 100 to 110 degrees for driving (Peli, 2002). An amount of 30Δ, typical in this population, would predict a field expansion of 17 degrees and would make most of them eligible for driving in nearly all US states.

### Limitations

We and others identified cases of strabismus that were visual field expanding. It is still unknown whether the visual field expansion is helpful for mobility or other uses. Head turn and tilt were only occasionally reported but seldom measured in these young patients. Thus, we did not investigate whether head turning was an accompanying behavioral compensation.

### Conclusions

Field expansion of just a few degrees in the middle peripheral field (55° eccentricity) is assumed to be helpful for monocular children turning their face towards the blinded eye.[27] Therefore, a few degrees in the central visual field is likely to be more beneficial. Nevertheless, some patients may prioritize cosmesis over driving. Clinicians should consider and discuss these issues before strabismus surgery in patients with HH.
